# The SETD8/ELK1/bach1 complex regulates hyperglycaemia-mediated EndMT in diabetic nephropathy

**DOI:** 10.1186/s12967-022-03352-4

**Published:** 2022-03-29

**Authors:** Xue Li, Lihong Lu, Wenting Hou, Fei Wang, Ting Huang, Zhipeng Meng, Minmin Zhu

**Affiliations:** 1 Department of Anesthesiology, Fudan University Shanghai Cancer Center; Department of Oncology, Shanghai Medical College, Fudan University, Shanghai, 200032 China; 2 Department of Anaesthesiology, Huzhou Hospital Affiliated to Zhejiang University, Affiliated Central Hospital of HuZhou University, Huzhou, 313000 Zhejiang China; 3 Department of Anesthesiology, Shanghai General Hospital, Shanghai Jiao Tong University School of Medicine, Shanghai, 200080 People’s Republic of China

**Keywords:** Diabetic nephropathy, SETD8, ELK1

## Abstract

**Background:**

Diabetic nephropathy (DN), the most common microvascular complication in patients with diabetes, induces kidney failure. Previous research showed that endothelial-to-mesenchymal transition (EndMT) of human glomerular endothelial cells (HGECs) is involved in the progression of DN. Moreover, SET domain-containing protein 8 (SETD8), ETS-domain containing protein (ELK1) and BTB and CNC homology 1 (bach1) all participate in endothelial injury. In this study, we hypothesize that the SETD8/ELK1/bach1 functional axis is involved in mediating EndMT in diabetic nephropathy.

**Methods:**

Immunohistochemistry, Western blotting and qPCR were performed to determine the protein and mRNA levels of genes in HGECs and the kidney tissues of participants and rats. Immunofluorescence, Co-IP and GST pulldown assays were performed to verify the direct interaction between SETD8 and ELK1. ChIP and dual-luciferase assays were performed to determine the transcriptional regulation of bach1 and Snail. AVV-SETD8 injection in rat kidney was used to verify the potential protective effect of SETD8 on DN.

**Results:**

Our current study showed that hyperglycaemia triggered EndMT by increasing Snail expression both in vitro and in vivo. Moreover, high glucose increased bach1 expression in HGECs, positively regulating Snail and EndMT. As a transcription factor, ELK1 was augmented and participated in hyperglycaemia-induced EndMT via modulation of bach1 expression. Moreover, ELK1 was found to associate with SETD8. Furthermore, SETD8 negatively regulated EndMT by cooperating with bach1 to regulate Snail transcription. Furthermore, histone H4-Lys-20 monomethylation (H4K20me1), which is downstream of SETD8, was accompanied by ELK1 localization at the same promoter region of bach1. ELK1 overexpression enhanced bach1 promoter activity, which disappeared after specific binding site deletion. Mutual inhibition between ELK1 and SETD8 was found in HGECs. In vivo, SETD8 overexpression decreased ELK1 and bach1 expression, as well as EndMT. Moreover, SETD8 overexpression improved the renal function of rats with DN.

**Conclusions:**

SETD8 cooperates with ELK1 to regulate bach1 transcription, thus participating in the progression of DN. In addition, SETD8 interacts with bach1 to modulate Snail transcription, thus inducing EndMT in DN. SETD8 plays a core role in the SETD8/ELK1/bach1 functional axis, which participates in hyperglycaemia-mediated EndMT in DN, and SETD8 may be a potential therapeutic target for DN.

*Trial registration* ChiCTR, ChiCTR2000029425. 2020/1/31, http://www.chictr.org.cn/showproj.aspx?proj=48548

**Supplementary Information:**

The online version contains supplementary material available at 10.1186/s12967-022-03352-4.

## Introduction

Diabetic nephropathy (DN), which is regarded as the common and primary microvascular complication of diabetes mellitus, has been proven to induce terminal-stage renal diseases [1, 2]. Glycated haemoglobin (HbA1c), as an indicator of long-term glycaemic control, has been proposed as a screening target for diabetic nephropathy and is also a glycaemic marker in patients with gestational diabetes mellitus and advanced chronic kidney disease [3–6]. In addition to HbA1c, the clinical manifestations of diabetic nephropathy include a gradual increase in urinary albumin and a decrease in the glomerular filtration rate [7, 8]. Currently, if DN develops into renal failure, the patient’s treatment costs and mortality will increase exponentially, and few effective treatment approaches for DN are available [9, 10]. Therefore, the search for the underlying mechanism of DN has practical value.

A recent report found that endothelial-to-mesenchymal transition (EndMT) occurs in glomerular endothelial cells, which is important for the progression of DN [11, 12]. EndMT occurs in the glomerular endothelium of patients with DN, as shown by a decrease in CD31 but an increase in α-SMA expression [13]. In EndMT, endothelial cells develop mesenchymal characteristics, replacing the endothelial phenotype [14], which is a specific type of epithelial-mesenchymal transition (EMT). Its representative genes include Snail, α-SMA, vimentin and CD31. Among these genes, Snail is a critical transcriptional regulator of EndMT [15]. Thus, EMT modulators may be involved in the regulation of EndMT by changing the expression of Snail [16].

Previous reports revealed that Bric-a-brac/Tramtrack/Broad (BTB) and cap'n'collar (CNC) homology 1 (bach1) impairs angiogenesis and mediates oxidative stress in vascular endothelial cells [17, 18]. In addition, bach1 participates in EMT in cancer cells [19]. However, the mechanism by which bach1 participates in hyperglycaemia-mediated EndMT has not yet been studied.

Moreover, some studies have demonstrated that ETS domain-containing protein (ELK1), a member of the E26 transformation-specific (ETS) oncogene family, is involved in the modulation of cell proliferation, apoptosis, differentiation and tumorigenesis [20–25]. Moreover, ELK1 was reported to participate in oxidized low-density lipoprotein-induced endothelial cell apoptosis [24]. ELK1 plays an important role in transforming growth factor-beta-induced EndMT [26, 27]. However, the role of ELK1 in hyperglycaemia-induced EndMT is unclear.

In addition to the above, SETD8, as the sole nucleosome-specific methyltransferase, can regulate the monomethylation of histone H4 lysine 20 (H4K20me1) [28]. Our previous studies demonstrated that suppression of SETD8 aggravates high glucose-induced vascular endothelial injury [29–31]. In addition, SETD8 was reported to mediate EMT [32]. At present, no research has shown that SETD8 participates in high glucose-induced EndMT.

The present study showed that SETD8 interacts with bach1 to regulate the transcription of Snail, leading to the occurrence of EndMT, which is involved in the progression of DN. In addition, SETD8 cooperates with ELK1 to regulate the transcription of bach1 by affecting histone methylation at the promoter region of bach1. Thus, SETD8 plays a key role in the progression of DN, and can be employed as the new target in the treatment of DN.

## Methods

### Subjects

This experiment was approved by the Ethics Committee of Huzhou Central Hospital (licence number: 20191209-01) and followed the Declaration of Helsinki. The present study recruited thirty patients with DN and type 2 diabetes. Additionally, thirty diagnosed renal cancer patients with normal renal function served as controls. All the participants signed informed consent forms. Exclusive criteria include advanced liver disease, renal failure, stroke, and other cardiovascular diseases.

### Rat model of DN

Under the provisions of the Guide for the Care and Use of Laboratory Animals of Fudan University Shanghai Cancer Center and the Guide for the Care and Use of Laboratory Animals published by the US NIH (2011), male SD rats weighing 300–400 g were employed. The animals were kept in a temperature-controlled environment (22 °C to 25 °C) and maintained in a 12-h light/dark cycle. All rats underwent unilateral nephrectomy (Unx) under isoflurane anaesthesia (3–4% induction, 1.5–2.5% maintenance, 100% oxygen) and were sent back to the care facility for 9 weeks. Three weeks after Unx, the rats that received a single intraperitoneal injection of citrate buffer (0.1 M, pH 4.5) were defined as the control group (con). Rats treated with a high-sugar and high-fat diet for 9 weeks after Unx and intraperitoneal injection of streptozotocin (STZ, 50 mg/kg) 3 weeks after Unx were defined as the DN group (n = 10) [33]. To clarify the protective effect of SETD8 overexpression in DN, we injected the rats with DN with AVV-SETD8 or control vectors into the contralateral kidney at the time of Unx. The rats were defined as the DN-AVV group (n = 10) and DN-AVV-SETD8 group (n = 10) accordingly [34, 35].

### Immunohistochemistry (IHC)

Tissue slides were deparaffinized and then stored in methanol containing 3% hydrogen peroxide. After the background was blocked, the slides were incubated with anti-SETD8 (dilution 1:200, ProteinTech, Wuhan, China), anti-ELK1 (dilution 1:200, ProteinTech), anti-bach1 (dilution 1:200, ProteinTech, anti-Snail (dilution 1:200, ProteinTech,), anti-vimentin (dilution 1:200, ProteinTech), anti-α-SMA (dilution 1:200, ProteinTech), and anti-CD31 (dilution 1:200, Abcam, Cambridge, UK) antibodies at 4 °C overnight. The next day, the slides were cultured with secondary antibodies at 37 °C. Finally, a DAB Detection Kit (GeneTech, Shanghai, China) was applied to stain the slides, and haematoxylin was used for counterstaining.

### Cell culture and intervention

HGECs were procured from Procell (Wuhan, China) and incubated with 5 mM glucose and 10% foetal bovine serum in an incubator at 37 °C in a humidified 5% carbon dioxide atmosphere. Cells were cultivated in high glucose (25 mM) DMEM for 3 days for the high glucose treatment. The apoptosis control used glucose (5 mM) mixed with mannitol (20 mM).

### Western blot

The samples were extracted from different cell groups and boiled with loading buffer for 10 min. The proteins were separated by 8–10% SDS-PAGE. The PVDF membranes were cultured with primary antibodies at 4 °C overnight. The primary antibodies were antibodies against β-actin (Dilution 1:2000, ProteinTech), SETD8, ELK1, bach1, Snail, vimentin, α-SMA, CD31 and H4K20me1 (Dilution 1:1000, Abcam). The next day, the membranes were cultured with the secondary antibodies. After that, the membranes were detected by the ECL system.

### Quantitative real-time PCR (qPCR)

Hieff UNICON® qPCR TaqMan Probe Master Mix (Yeasen, Shanghai, China) was used to perform quantitative qPCR to determine the target genes. The primer sequences are shown in Additional file 1: Table S1. The relative gene expression was calculated by the 2^−△△CT^ method. Data are shown as the fold change relative to the control group. In addition, the ratio of the control group was set as 1.

### Coimmunoprecipitation (Co-IP)

Cell protein lysates were isolated with lysis buffer, which was mixed with primary antibodies against bach1, SETD8, ELK1 and IgG at 4 °C overnight for endogenous IP. The next day, the lysates were incubated with 50 μL of protein Dynabeads (Thermo, MA, USA) for 6 h at 4 °C. Furthermore, the beads were washed with RIPA 3 times to remove impurities. Finally, 20 µL of IP lysates was added to 2× loading buffer and boiled together. The results were analysed by Western blots.

### Immunofluorescence assay

After HGECs were seeded onto glass slides, the cells were fixed with 4% paraformaldehyde for 10 min. The cells were incubated with anti-SETD8 and anti-ELK1 antibodies at 4 °C overnight. Then, they were sequentially incubated with fluorescent secondary antibodies for 1 h at 37 °C. Next, DAPI (Yeasen) was used to stain nuclei. Finally, a confocal fluorescence microscope (Leica) was employed to capture images.

### GST pulldown assay

We purchased His-SETD8 (ProteinTech) and GST-ELK1 (ProteinTech) fusion proteins for the experiment. The fusion proteins were mixed for 12 h at 4 °C in GST binding buffer. Anti-His or anti-GST beads were added and incubated with the fusion protein for an additional 4 h. The beads were washed three times, and the proteins were detected by western blotting.

### siRNA treatments

In the experiment, HGECs were transfected with siRNA against ELK1 and bach1 using Lipofectamine 3000. ELK1 siRNA (Biotend) sequences were as follows: siRNA-a, 5′-GGUACUACUAUGACAAGAAdTdT-3′ and siRNA-b, 5′-GCAGCUGCUGAGAGAGCAAdTdT-3′. The bach1 siRNA sequences were as follows: siRNA-a, 5′-CAGACAUAUGAGUCCAUGUdTdT-3′ and siRNA-b, 5′-CAGCAAUUUAACAGCUUGAdTdT-3′.

### Short hairpin RNA (shRNA) and mutant SETD8

SETD8 shRNAs and mutant SETD8^R295G^ plasmid [27] were transfected into HGECs. The sequences of shRNA were as follows: shRNA-a, 5′-CAACAGAATCGCAAACTTA-3′ and shRNA-b, 5′-CAACAGAATCGCAAACTTA-3′.

### Chromatin immunoprecipitation (ChIP) assay

The ChIP assay kit (Millipore, MA, USA) was used in the study. Briefly, cells (1 × 10^7^) were settled with 1% formaldehyde. Then, glycine (2.5 M) was added to stop the crosslinking reaction. Chromatin was subjected to ten ultrasounds. After centrifugation, the supernatant was incubated with anti-bach1, anti-ELK or anti-H4K20me1 antibodies and IgG at 4 °C. Agarose beads were applied to connect with immunoprecipitants. Furthermore, DNA–protein crosslinking was reversed by using a water bath at 65 °C for 6 h. The enriched sequences of the purified DNA were analysed by PCR. The bach1 oligonucleotide primer sequences were forward 5′-ACTGGCTCAAGGTGGAAGGA-3′, and reverse 5′-CAGGCTGCCTCAGTTCATGG-3′. The Snail oligonucleotide primer sequences were forward 5′-TAAATTGACACGGGACGGGG-3′, and reverse 5′-CTGGTTCTAGCTGGAGAGCG-3′. Furthermore, a re-ChIP assay was performed to verify whether SET8 and ELK1 occupied the same binding site on the bach1 promoter region. The chromatin from the beads was eluted by 10 mM DTT after the standard ChIP procedure. The eluent was then diluted with sonication buffer before undergoing the ChIP process again.

### Dual-luciferase assay

The Dual-luciferase Assay Kit was employed to measure the impact of ELK1 on the bach1 promoter. The bach1 promoter was amplified from genomic DNA of HGECs and ligated into the pGL3-Basic vector. Moreover, the deleted promoter site was constructed for comparison. Then, pGL3-DAPK3 and pGL3-DAPK3^Del^ were transfected into HGECs. The relative luciferase activity was used to evaluate the influence of ELK1 on bach1 promoter activity.

### Statistical analysis

In this study, the sample size of the in vivo experiment was 10, the sample size of the in vitro experiment was 5, and statistical significance was obtained.

The data from separate experiments were analysed by GraphPad Prism 8 Project software. The comparison of means of two groups was performed by two-tailed unpaired t tests. We used a one-way ANOVA test to compare the means of more than 2 groups. p < 0.05 was considered statistically significant, and the data were plotted using GraphPad Prism 8.

## Results

### Development of EndMT and increased expression of bach1 in DN

**Fig. 1:**
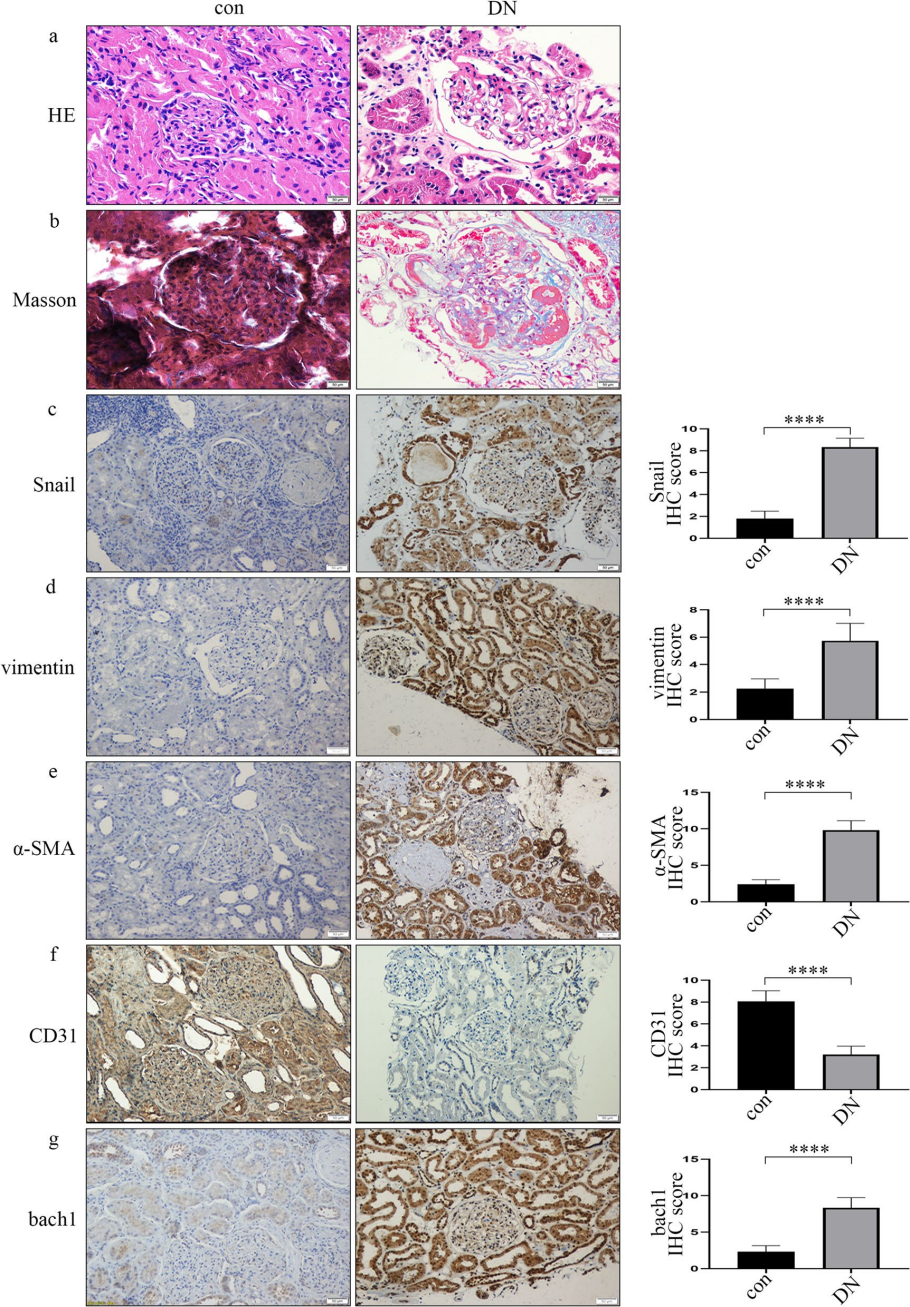
Development of EndMT and increased expression of bach1 in DN. **a** Representative images of HE staining of renal biopsy specimens from the DN group and the control group. The result conveyed that the basement membrane of glomerular capillaries thickened, and mesangium proliferated, thereby forming the nodular sclerosis (n = 30/group, scale bar: 50 μm). **b** Representative images of Masson’s trichrome staining of renal biopsy specimens from the DN group and the control group. Masson trichrome staining exhibited a blue colour in DN patient samples, representing collagen deposition and interstitial fibrosis (n = 30/group, scale bar: 50 μm). **c**–**g** The IHC results of different genes in renal biopsy specimens of the DN group and the control group. Among them, the expression of Snail, vimentin, α-SMA and bach1 was up-regulated, and the expression of CD31 was down-regulated. (n = 30/group, scale bar: 50 μm)

The clinical information of the participants and rats is shown in Table 1. It has been reported that EndMT of glomerular endothelial cells participates in DN [11, 12]. The HE staining of patient samples is shown in Fig. 1a. The result conveyed that the basement membrane of glomerular capillaries thickened, and mesangium proliferated, thereby forming the nodular sclerosis. Masson trichrome staining exhibited a blue colour in DN patient samples, representing collagen deposition and interstitial fibrosis (Fig. 1b). As the key regulator of EndMT, Snail expression was upregulated in the patients with DN (Fig. 1c). Next, we detected EndMT genes in the DN group and the control group. Compared to that of the controls, the expression of CD31 was suppressed in glomerular endothelial cells of the patients with DN, while vimentin and α-SMA expression increased (Fig. 1d–f). Similarly, representative images of EndMT were also found in the rats with DN (Additional file 2: Fig. S1a–f). According to previous reports, bach1 is involved in EMT in cancer cells [19]. Similarly, we found that the bach1 expression level was increased in the glomerular endothelium in DN (Fig. 1g, Additional file 2: Fig. S1g). Moreover, Western blot and qPCR results indicated that the levels of bach1, Snail, vimentin and α-SMA in the kidneys of the rats with DN were higher than those in the kidneys of the controls (Additional file 3: Fig. S2a–d, f), while the levels of CD31 were lower (Additional file 3: Fig. S2a, e).

**Table 1:** Various indicators of participants and rats in the control (con) and diabetic nephropathy (DN) groups

Human			
Variables	Con	DN	P-value
Male (%)	50	50	0
Age (years)	51.3 ± 8.2	52.6 ± 9.0	0.83
BMI (kg/m^2^)	22.5 ± 1.1	23.0 ± 3.3	0.35
SBP (mmHg)	107.4 ± 16.3	144.9 ± 21.6	< 0.0001
DBP (mmHg)	61.2 ± 8.8	79.2 ± 12.6	< 0.0001
HbA1C (%)	5.2 ± 1.7	7.4 ± 0.7	0.0146
FBS (mmol/L)	4.3 ± 1.1	9.5 ± 0.8	< 0.0001
CREA (µmol/L)	79.6 ± 26.7	227.8 ± 95.6	< 0.0001
ALB (g/L)	46.6 ± 9.4	30.6 ± 5.4	0.0049
CCr (mL/min)	95.6 ± 8.7	54.9 ± 30.1	< 0.0001
24 h UTP (mg)	98.2 ± 10.1	3494.9 ± 1883.8	< 0.0001
UA (µmol/L)	187.3 ± 28.7	404.8 ± 83.3	< 0.0001
TP (g/L)	65.0 ± 4.7	52.1 ± 4.8	0.0008

Rats			
Variables	Con	DM	P-value
Weight (g)	356.9 ± 27.8	533.0 ± 41.1	< 0.0001
Weight of kidney (g)	1.6 ± 0.2	2.7 ± 0.7	0.0001
FBS (mmol/L)	5.3 ± 0.8	11.6 ± 1.8	< 0.0001
CREA (µmol/L)	5.0 ± 1.1	11.6 ± 1.0	< 0.0001
UREA (µmol/L)	6.7 ± 0.6	11.5 ± 1.4	< 0.0001
UMP (mg/L)	115.8 ± 13.0	171.5 ± 11.0	< 0.0001

Data are presented as the means ± standard deviation, *p < 0.05, n = 30 per human group, n = 10 per rat group. Statistical analysis was carried out by a Student’s t test

*BMI* body mass index, *SBP* systolic blood pressure, *DBP* diastolic blood pressure, *HbA1c* glycated hemoglobin, *FBS* fasting blood sugar, *CREA* creatinine, *ALB* albumin, *CCr* creatnine clearance, *24 h UTP* uric total protein, *UA* uric acid, *TP* total protein, *UMP* urine micro-protein

### Bach1 is required for EndMT in HGECs under hyperglycaemic conditions

**Fig. 2:**
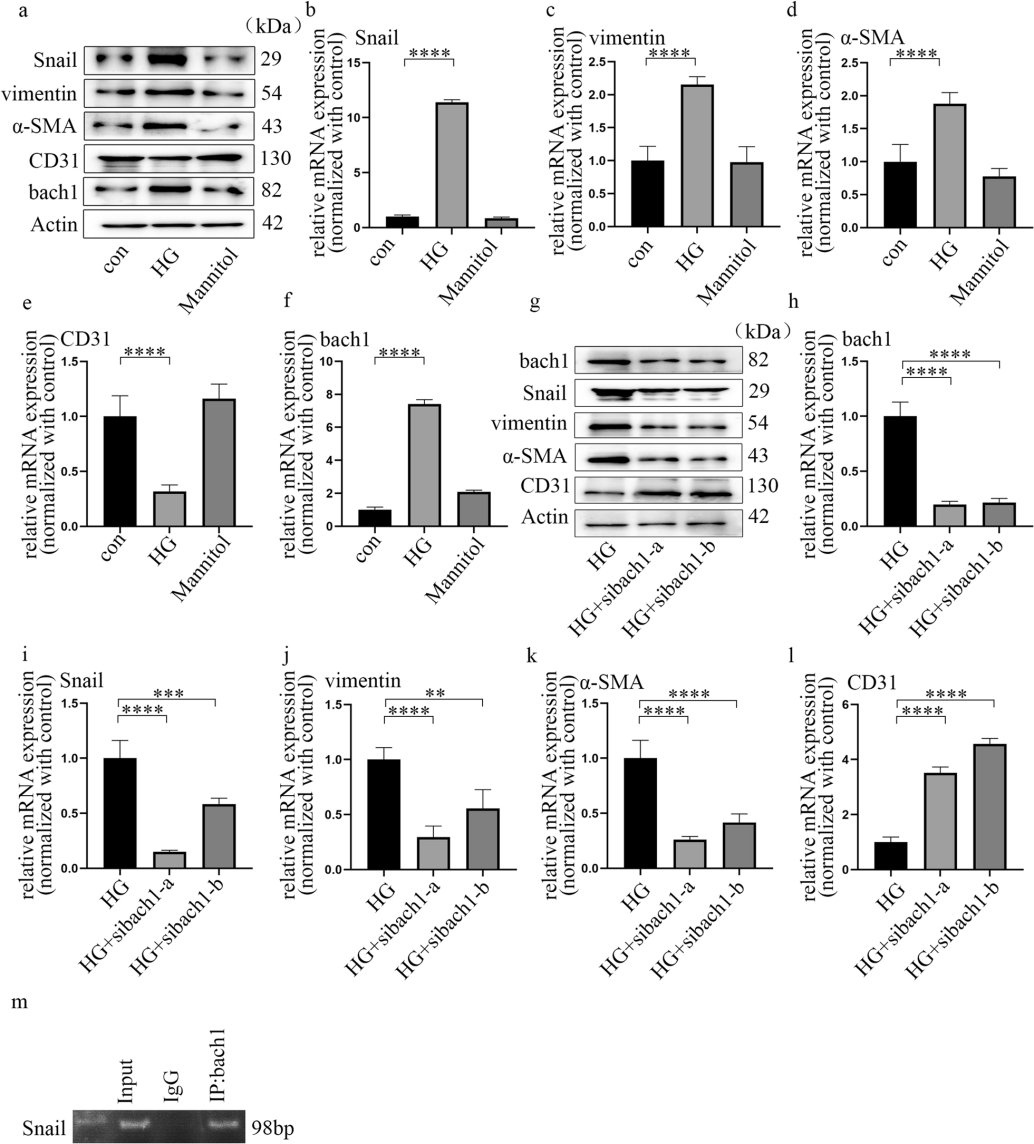
Bach1 is required for EndMT in HGECs under hyperglycaemic conditions. **a** Western blot results of genes in different cell groups. **b**–**f** mRNA expression of genes in different cell groups (n = 5/group). **g** Western blot results of genes in different cell groups. **h**–**l** mRNA expression of genes in different cell groups (n = 5/group). **m** ChIP results showed that bach1 accumulated at the Snail promoter region. (Data are presented as the mean ± standard deviation, *p < 0.05, **p < 0.01, ***p < 0.001, ****p < 0.0001, statistical analysis was carried out by one-way ANOVA.)

HGECs were used to explore whether bach1 modulates EndMT in DN. Cells were cultured in normal glucose for three days or high glucose for three days to further confirm that EndMT was induced under hyperglycaemic conditions and to determine the role of bach1. The results showed that a high glucose environment suppressed CD31 expression while increasing Snail, vimentin and α-SMA expression in HGECs (Fig. 2a–e). Mannitol treatment had no effect on these trends. According to previous research, bach1 participates in EMT in cancer cells [19]. Therefore, we investigated bach1 expression in HGECs. The results indicated that hyperglycaemia increased both the protein (Fig. 2a) and mRNA levels (Fig. 2f) of bach1 in HGECs. In this study, two independent siRNAs targeting bach1 were used to further explore whether bach1 is involved in EndMT induced by high glucose in HGECs, and the effects of si-bach1 were confirmed (Fig. 2g, h). Our data revealed that si-bach1 reversed CD31 suppression and decreased the levels of Snail, vimentin and α-SMA under hyperglycaemic conditions (Fig. 2g, i–l). This finding indicated that bach1 promoted EndMT in high glucose-treated HGECs. Moreover, the association between bach1 and SETD8 was determined by Co-IP (Additional file 3: Fig. S2g). Further experiments also demonstrated that SETD8 knockout in HGECs enhanced the expression of Snail to promote EndMT progression (Additional file 3: Fig. S2h–m). Moreover, bach1 was accompanied by SETD8 binding to the promoter region of Snail, which may directly regulate the transcriptional activity of Snail (Fig. 2m; Additional file 3: Fig. S2n, o). These data may indicate that bach1 interacted with SETD8 to alter Snail expression, thereby inducing EndMT in high glucose-cultured HGECs.

### ELK1 participated in EndMT by augmenting bach1 expression in high glucose-cultured HGECs

**Fig. 3:**
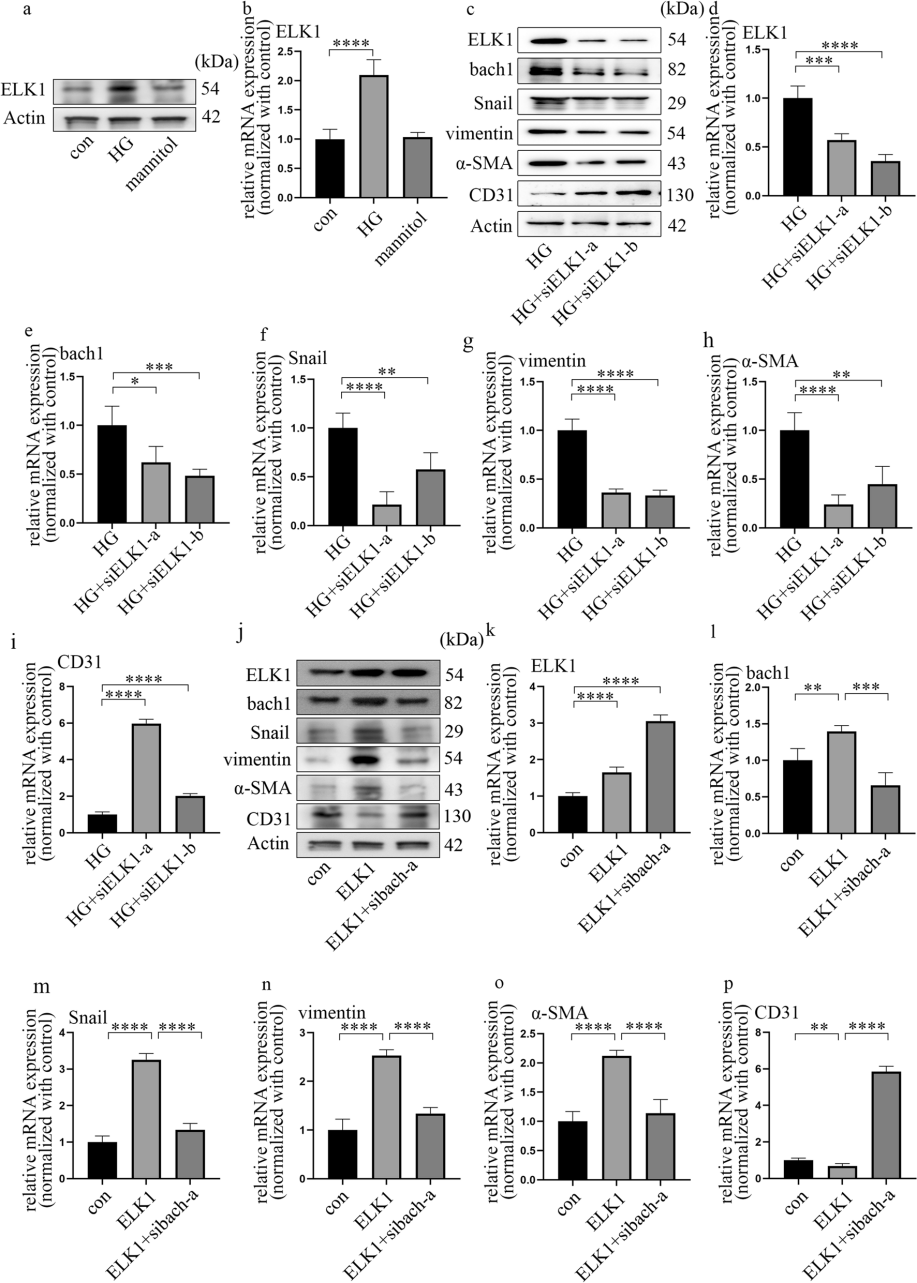
ELK1 participated in EndMT by augmenting bach1 expression in high glucose-cultured HGECs. **a** Western blot results of ELK1 in different cell groups. **b** mRNA expression of ELK1 in different cell groups (n = 5/group). **c** Western blot results of genes in different cell groups. **d**–**i** mRNA expression of genes in different cell groups (n = 5/group). **j** Western blot results of genes in different cell groups. **k**–**p** mRNA expression of genes in different cell groups (n = 5/group, data are presented as the mean ± standard deviation, *p < 0.05, **p < 0.01, ***p < 0.001, ****p < 0.0001, statistical analysis was carried out by one-way ANOVA)

As an important transcription factor, ELK1 expression was increased under high-glucose conditions (Fig. 3a, b; Additional file 4: Fig. S3a–d). This result suggested that ELK1 may also be involved in the regulation of EndMT in DN.

To explore the function of ELK1 on hyperglycaemia-induced bach1 and EndMT, we verified its effects by increasing and inhibiting the expression level of ELK1. Western blotting (Fig. 3c) and qPCR (Fig. 3d) demonstrated the effect of si-ELK1. In addition, si-ELK1 reversed bach1 expression and neutralized EndMT in high glucose-cultured HGECs (Fig. 3c, e–i). In addition to the findings above, our present study showed that ELK1 overexpression had the same effect as the hyperglycaemia treatment (Fig. 3j–p). To determine whether the effect of ELK1 overexpression was due to augmentation of bach1 expression, we silenced bach1 in the ELK1-overexpressing HGECs. The data proved that bach1 silencing reversed the EndMT caused by ELK1 overexpression in HGECs (Fig. 3j–p). The results showed that ELK1 overexpression promoted bach1 expression, thereby inducing EndMT in the HG-cultured HGECs.

### ELK1 is related to SETD8

**Fig. 4:**
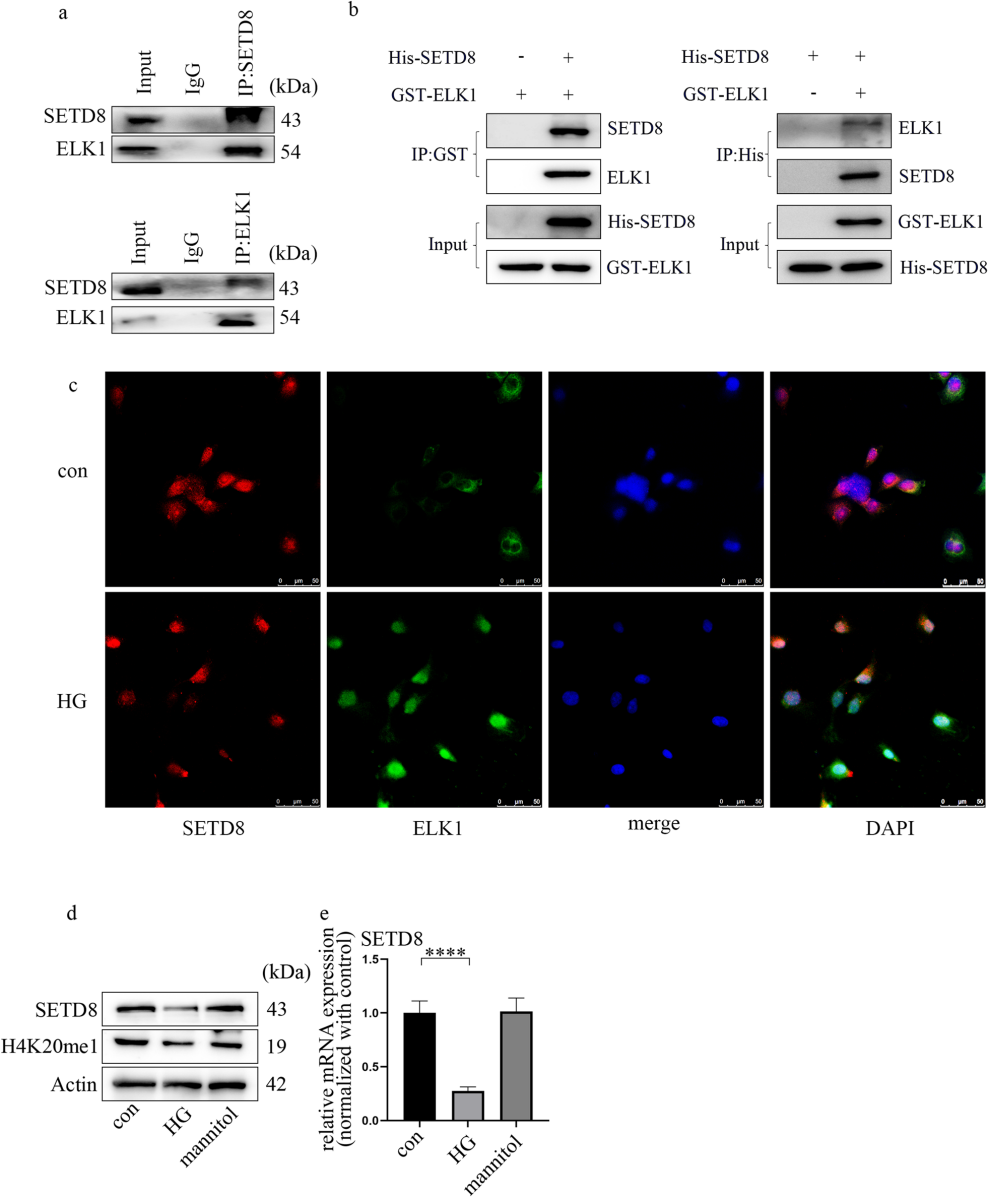
ELK1 is related to SETD8. **a** Co-IP verified the connection between ELK1 and SETD8 in HGECs. **b** GST pulldown verified the direct connection between ELK1 and SETD8 in HGECs. **c** Immunofluorescence assays illustrated the nuclear translocation of ELK1 under HG environment. Further, the result verified the colocalization of ELK1 and SETD8 in HGECs (scale bar: 50 μm). **d** Western blot results of genes in different cell groups. **e** mRNA expression of genes in different cell groups (n = 5/group, data are presented as the mean ± standard deviation, *p < 0.05, **p < 0.01, ***p < 0.001, ****p < 0.0001, statistical analysis was carried out by one-way ANOVA)

To clarify the internal mechanism by which ELK1 regulates bach1 expression and EndMT in HGECs, we used bioinformatics to predict the proteins associated with ELK1. There are various kinds of proteins directly or indirectly associated with ELK1, including SETD8 (Additional file 5: Fig. S4a). The enrichment analysis of GO pathways showed the three main parts, which are shown in Additional file 5: Fig. S4b. The terms included histone methyltransferase activity. Our previous research corroborated that SETD8 suppression aggravates vascular endothelial injury under hyperglycaemia [29–31]. In addition, SETD8 mediates the occurrence of EMT. Moreover, the Co-IP results (Fig. 4a) verified the cooperation between ELK1 and SETD8 in the HGECs. The direct interaction between ELK1 and SETD8 was confirmed by a GST pulldown assay (Fig. 4b). Immunofluorescence analysis confirmed that SETD8 and ELK1 were colocalized in GECs (Fig. 4c), and high glucose clearly mediated ELK1 nuclear translocation (Fig. 4c). Furthermore, our data revealed that hyperglycaemia inhibited SETD8 (Fig. 4d, e) levels in HGECs. Consistently, H4K20me1, a downstream target of SETD8, was also inhibited under hyperglycaemic conditions (Fig. 4d). This trend of SETD8 was also confirmed in the rats with DN and patients (Additional file 5: Fig. S4c–f).

### Suppression of SETD8 regulated hyperglycaemia-induced EndMT by enhancing bach1 expression in HGECs

**Fig. 5:**
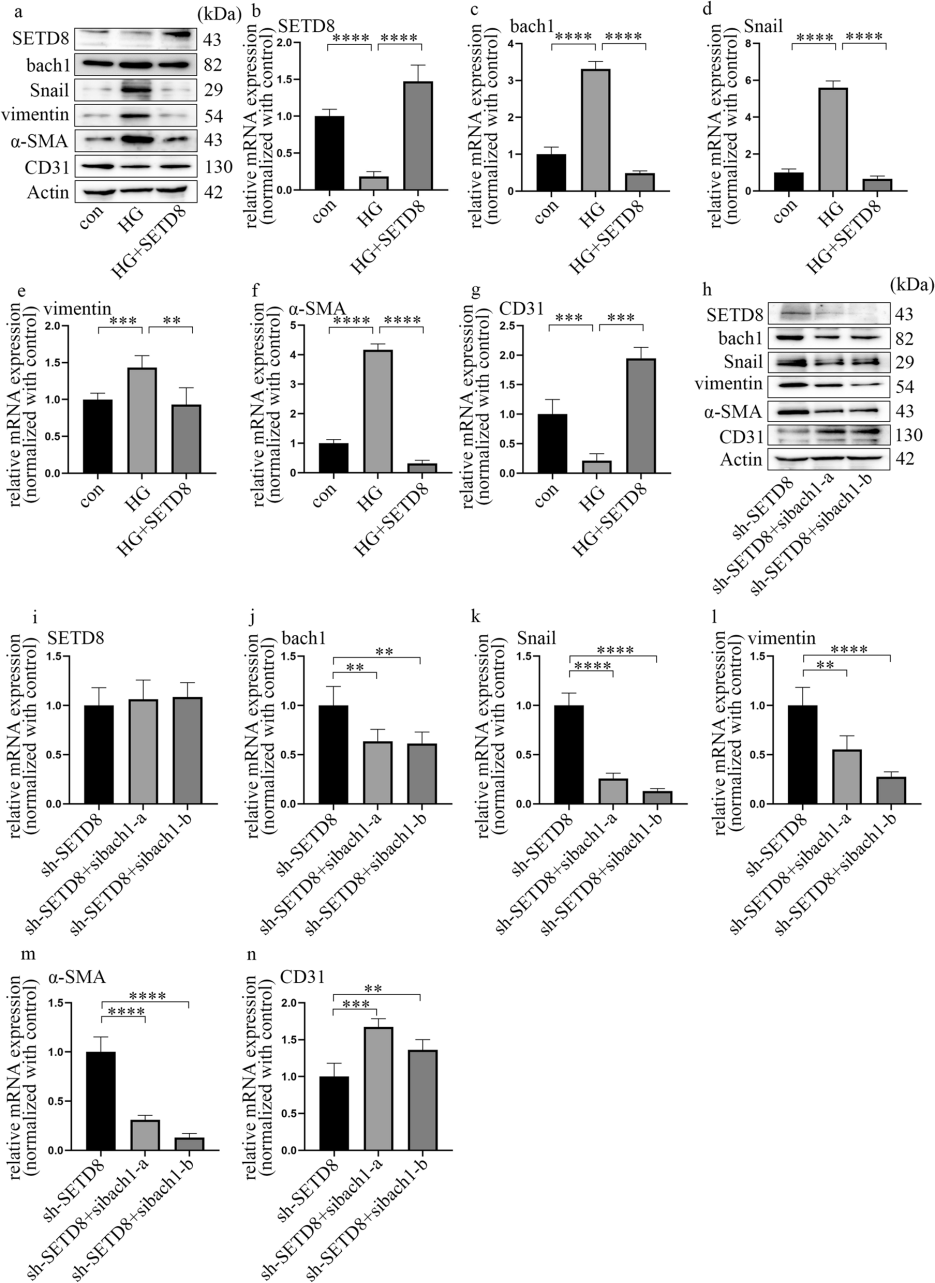
Suppression of SETD8 regulated hyperglycaemia-induced EndMT by enhancing bach1 expression in HGECs. **a** Western blot results of genes in different cell groups. **b**–**g** mRNA expression of genes in different cell groups (n = 5/group). **h** Western blot results of genes in different cell groups. **i**–**n** mRNA expression of genes in different cell groups (n = 5/group, data are presented as the mean ± standard deviation, *p < 0.05, **p < 0.01, ***p < 0.001, ****p < 0.0001, statistical analysis was carried out by one-way ANOVA)

To confirm the effect of SETD8 on bach1 expression and EndMT in high glucose-cultured HGECs, we used both loss of function and gain of function experiments. The overexpression of SETD8 was determined by western blotting (Fig. 5a) and qPCR (Fig. 5b). The data demonstrated that SETD8 overexpression reversed hyperglycaemia-induced bach1 expression (Fig. 5a, c). Additionally, SETD8 overexpression neutralized the reduction in CD31 (Fig. 5a, g) and the increase in Snail, vimentin and α-SMA (Fig. 5a, d–f) expression in HGECs in a high-glucose environment.

Moreover, the efficacy of sh-SETD8 was verified (Additional file 6: Fig. S5a, b). The effect of sh-SETD8 on bach1 expression and EndMT was similar to that of high glucose treatment (Additional file 6: Fig. S5a, c–g). To determine whether the effect of sh-SETD8 was due to increased expression of bach1, we silenced bach1 based on the inhibition of SETD8. The results indicated that bach1 silencing neutralized SETD8 silencing-induced EndMT in HGECs (Fig. 5h–n). All of these data revealed that SETD8 suppression enhanced bach1 expression in hyperglycaemic HGECs and is thus involved in the EndMT of DN.

### The transcriptional activity of bach1 is jointly regulated by ELK1 and SETD8 in HGECs

**Fig. 6:**
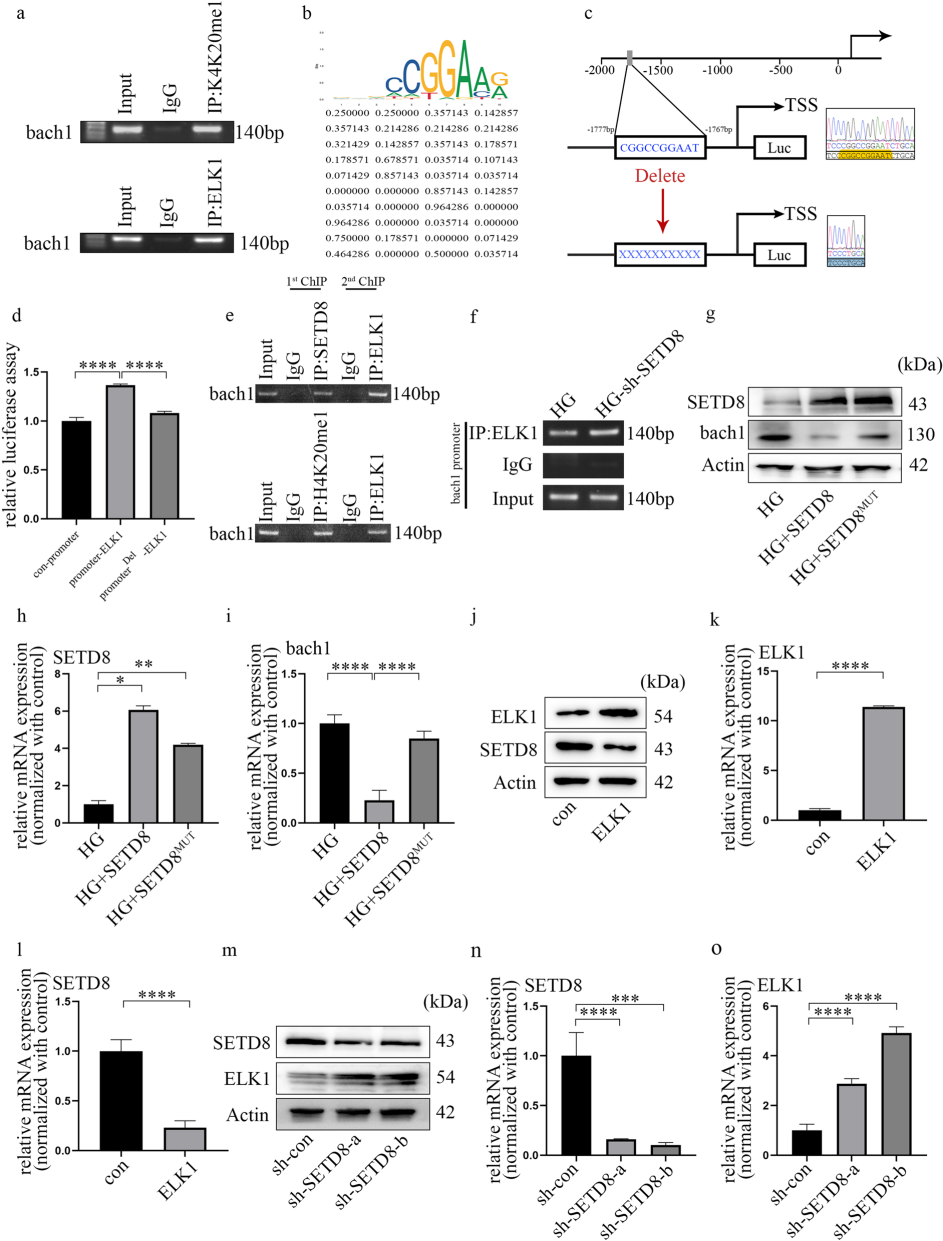
The transcriptional activity of bach1 is jointly regulated by ELK1 and SETD8 in HGECs. **a** ELK1 and H4K20me1 accumulated at the bach1 promoter region. **b** Schematic diagram of the bach1 promoter region containing an E-box and the conserved ELK1 binding site. **c**, **d** Bach1 promoter activity was determined by luciferase reporter assays after treatment. **e** ELK1 and SETD8, as well as K4K20me1, were located on the same promoter region of bach1 in HGECs. **f** The binding of ELK1 to the promoter region of bach1 was regulated by SETD8 in HGECs. **g** Western blot results of genes in different cell groups. **h**, **i** mRNA expression of genes in different cell groups (n = 5/group). **j** Western blot results of genes in different cell groups. **k**, **l** mRNA expression of genes in different cell groups (n = 5/group). **m** Western blot results of genes in different cell groups. **n**, **o** mRNA expression of genes in different cell groups (n = 5/group, data are presented as the mean ± standard deviation, *p < 0.05, **p < 0.01, ***p < 0.001, ****p < 0.0001, statistical analysis was carried out by one-way ANOVA)

Next, to determine whether ELK1 and SETD8 target bach1, we determined the genome-wide distribution of ELK1 and H4K20me1 in HGECs through ChIP analysis. The results showed that both ELK1 and H4K20me1 targeted the promoter of bach1 (Fig. 6a). The putative binding site of ELK1 is presented in Fig. 6b, c. Additionally, Fig. 6d shows that overexpression of ELK1 enhanced bach1 promoter activity, which could be reversed by deletion of the binding site. In the present study, re-ChIP was adopted to clarify whether the chromatin interaction between SETD8 and ELK1 was located at the same promoter region of bach1 (Fig. 6e). Moreover, we found increased occupation of ELK1 on the bach1 promoter region when SETD8 was silenced (Fig. 6f). In addition, SETD8^R259G^ (one SETD8 mutant) had no influence on bach1 transcription (Fig. 6g–i). These data indicated that ELK1 cooperated with SETD8 to affect the activity of the bach1 promoter region in the high glucose-treated HGECs. Moreover, SETD8-related H4K20me1 is essential for modulating bach1 expression in HGECs. Furthermore, overexpression of ELK1 decreased SETD8 expression (Fig. 6j–l). Consistently, sh-SETD8 augmented ELK1 expression in HGECs (Fig. 6m–o). Hence, these data indicated that ELK1 inhibits SETD8 in HGECs and vice versa.

### Overexpression of SETD8 ameliorated the pathological process in rats with DN

**Fig. 7:**
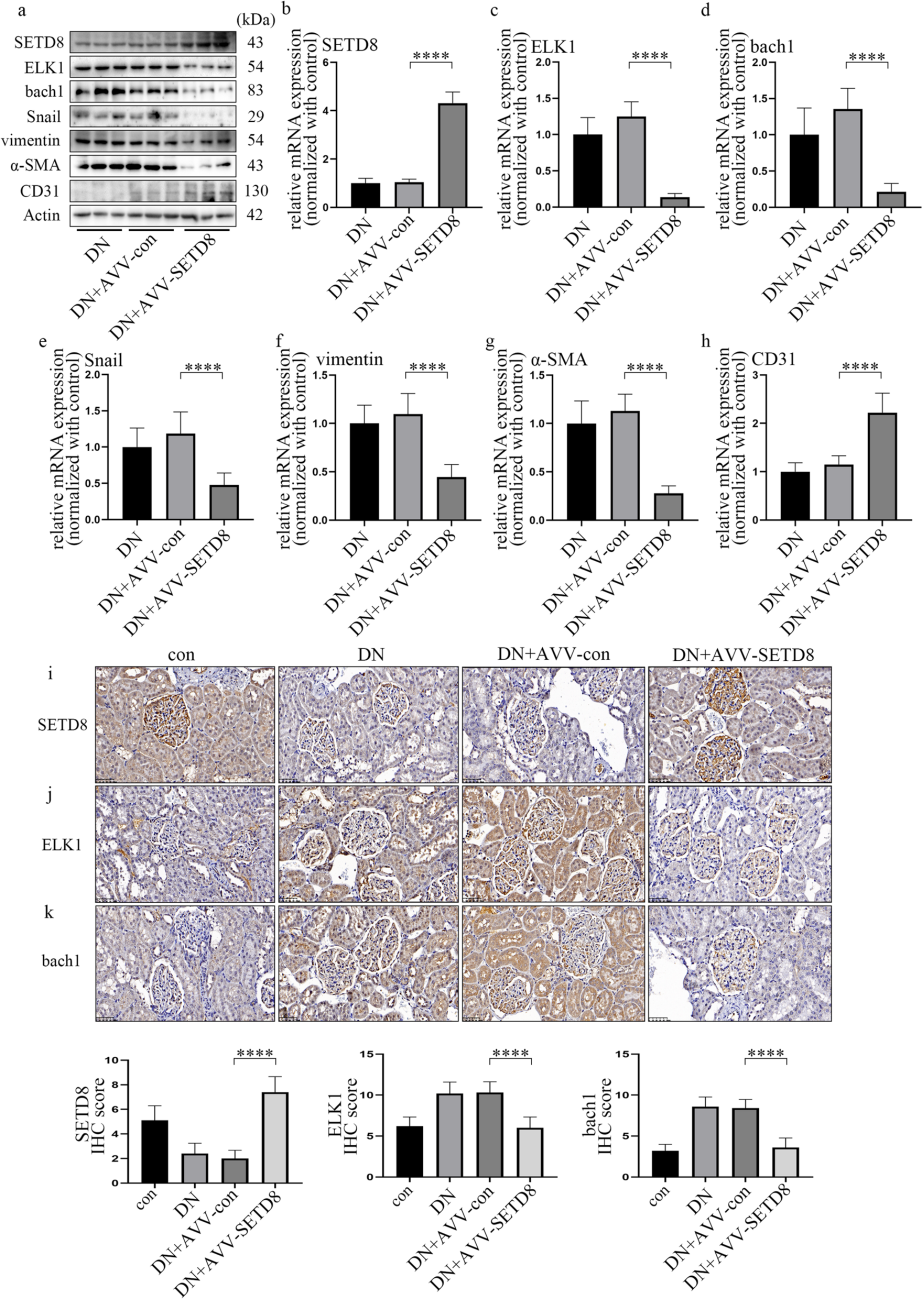
Overexpression of SETD8 ameliorated the pathological process in rats with DN. **a** Western blot results of genes in different rat groups. **b**–**h** mRNA expression of genes in different rat groups. (n = 5/group). **i**–**k** Immunostaining of different genes in the kidneys of rats with the corresponding treatments. Hyperglycaemia-induced upregulation of ELK1 and bach1 can be reversed by AVV-SETD8 overexpression (n = 10/group). (Data are presented as the mean ± standard deviation, *p < 0.05, **p < 0.01, ***p < 0.001, ****p < 0.0001, statistical analysis was carried out by one-way ANOVA.)

**Fig. 8:**
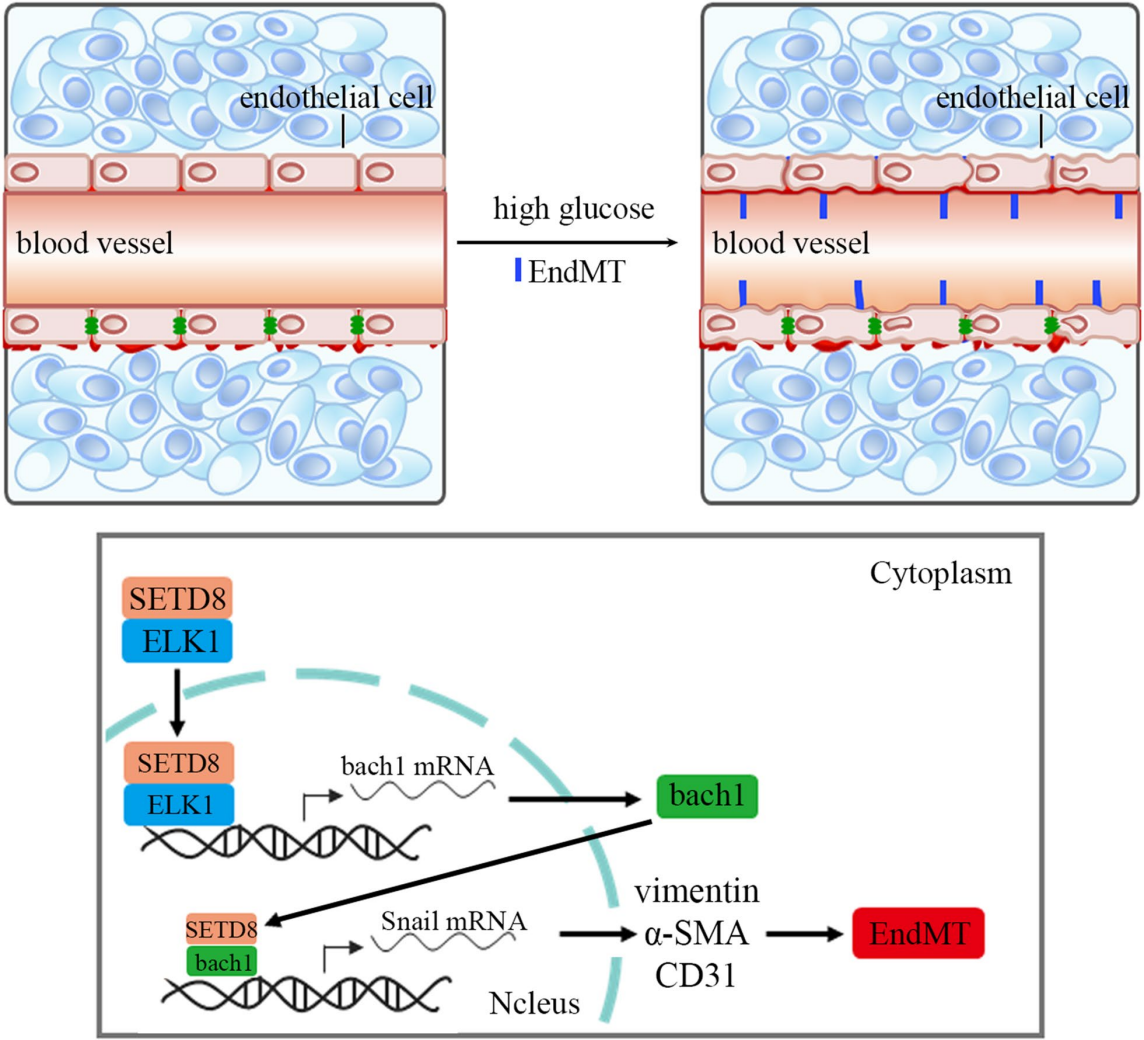
Schematic representation of the working model. SETD8 cooperates with ELK1 to regulate bach1 transcription, thus participating in the progression of DN. In addition, SETD8 interacts with bach1 to modulate Snail transcription, thus inducing EndMT in DN

To verify the protective effect of SETD8 overexpression in vivo, we used AVV-SETD8 in the experiments. The efficiency of AVV-SETD8 was tested by fluorescence microscopy, western blotting and qPCR (Additional file 7: Fig. S6a; Fig. 7a, b, i). Our data indicated that SETD8 overexpression decreased hyperglycaemia-induced ELK1 and bach1 expression, as well as EndMT in the kidneys (Fig. 7a, c–k; Additional file 7: Fig. S6b–e), and improved renal dysfunction in rats (Additional file 8: Fig. S7a–f). In conclusion, our study showed that SETD8 not only directly regulates the transcription of Snail but also associates with ELK1 to regulate bach1 expression, thus mediating EndMT in glomerular endothelial cells of patients and rats with DN (Fig. 8).

## Discussion

The novel findings of this research demonstrated that hyperglycaemia, by augmenting bach1 expression, participated in EndMT of glomerular endothelial cells. Moreover, SETD8 interacted with bach1 to regulate the transcriptional activity of Snail, which regulated the occurrence of EndMT. In addition, a high glucose environment enhanced ELK1 levels and restrained SETD8 expression. Meanwhile, SETD8 cooperated with ELK1 and occupied the bach1 promoter region at the same site, thereby regulating the transcription of bach1, thus mediating EndMT in hyperglycaemia-cultured HGECs.

EMT is a sophisticated cell phenotype reprogramming process that participates in organ injury [36]. Previous reports demonstrated that EMT plays a vital role in renal interstitial myofibroblasts, thus underpinning the progression of renal fibrosis [37]. Moreover, studies have found that EndMT is involved in the progression of DN [11, 12]. Similarly, our representative images of HE and Masson staining exhibited collagen deposition and fibrosis. Moreover, IHC staining illustrated that CD31 expression was suppressed, while Snail, vimentin and α-SMA levels were increased in the glomerular endothelial cells of DN. These data were in agreement with EndMT in kidney-aggravated DN [13]. Importantly, among these genes, Snail is the key regulator of EndMT in endothelial cells [38]. To determine whether EndMT in DN was due to a high glucose environment, we employed high glucose-cultured HGECs. The changes in the levels of Snail, CD31, vimentin and α-SMA confirmed that hyperglycaemia induced EndMT, and the result coincided with our previous study [39]. In addition, bach1 was verified to participate in EMT in cancer cells [19]. It was deduced that EMT and EndMT share cooperative modulators [16]. We next investigated whether bach1 was involved in regulating EndMT in hyperglycaemic HGECs. Bach1 levels were confirmed to be higher in DN. High glucose treatment increased bach1 expression and EndMT in HGECs, while inhibition of bach1 expression reversed these trends. ChIP assays suggested that bach1 can accumulate in the Snail promoter region. These data showed that upregulated bach1 expression is required for high glucose-mediated EndMT by regulating Snail in HGECs. Our previous studies indicated that SETD8 participates in hyperglycaemia-mediated increases in endothelial adhesion molecule expression [40], proinflammatory enzymes, proinflammatory cytokine production [29], and antioxidant imbalance [30], thus mediating vascular endothelial injury [29–31, 40]. In this study, our data indicated that SETD8 could regulate the expression of Snail. Co-IP indicated that SETD8 interacted with bach1 in HGECs. The ChIP assay confirmed that not only bach1 but also SETD8 occupied the Snail promoter. This finding indicated that SETD8 cooperated with bach1 to regulate Snail expression, thus participating in EndMT in DN.

ELK1 was reported to modulate cell proliferation, apoptosis, differentiation, and tumorigenesis [20–23, 25]. In addition, ELK1 mediates oxidized low-density lipoprotein-induced endothelial cell apoptosis [24]. Moreover, upregulated ELK1 expression has been confirmed to mediate cell death [20, 41]. Furthermore, ELK1 is activated by hypoxia in vascular endothelial cells [21, 42] and is involved in transforming growth factor-beta-induced EndMT [26, 27]. In this study, we found that high glucose augmented ELK1 expression in HGECs. Then, we investigated whether ELK1 regulated EndMT via modulation of bach1 in HGECs. We demonstrated that si-ELK1 reversed the upregulation of bach1 expression and EndMT under hyperglycaemic conditions. Moreover, overexpression of ELK1 aggravated bach1 levels and EndMT, which was neutralized by si-bach1. All these results indicated that ELK1 orchestrated EndMT in HG-cultured HGECs by augmenting bach1 expression.

To date, few reports have described the regulation of EndMT by SETD8. Indeed, it was reported that SETD8 participates in EMT by modulating TWIST [43] and cooperating with zinc finger E-box-binding homeobox 1 [32]. As a specific form of EMT, EndMT and EMT may possess shared modulators [16]. The present study confirmed that SETD8 overexpression neutralized high glucose-mediated upregulation of bach1 expression and EndMT in HGECs. Moreover, sh-SETD8 upregulated bach1 expression and mediated EndMT in HGECs, resulting in the same effect as high glucose treatment. Furthermore, si-bach1 neutralized sh-SETD8-mediated EndMT. Downstream of SETD8, H4K20me1 accumulates at the bach1 promoter region. All these findings indicated that high glucose-induced downregulation of SETD8 expression caused EndMT in HGECs by upregulating bach1 expression.

Previous reports have shown that epigenetic modifications can modulate the transcriptional activity of ELK1 [44, 45]. In this study, ELK1 was found to associate with SETD8, which is the sole nucleosome-specific methyltransferase. The results of the luciferase assay illustrated that upregulated ELK1 expression augmented bach1 promoter activity. Moreover, our data showed that SETD8 and ELK1 were located at the same promoter region of bach1 by the re-ChIP assay. Moreover, the occupancy of ELK1 on the bach1 promoter region was augmented in cells in which SETD8 was silenced. These data indicated that the transcriptional activity of ELK1 in DN was modulated by SETD8. Furthermore, SETD8 overexpression inhibited HG-induced bach1 expression, and the SETD8 mutant did not function. Our results indicated that SETD8-modulated H4K20me1 participates in the modulation of bach1 levels.

To further confirm the crucial and protective role of SETD8 in DN, we performed SETD8 overexpression experiments in vivo. Our data illustrated that the increase in ELK1 and bach1 expression and EndMT, as well as the decline in renal function, were reversed by SETD8 overexpression in in vivo experiments. Thus, the SETD8/ELK1/bach1 axis may be a potential therapeutic target for blocking the occurrence and development of EndMT in DN.

There are some limitations in our study. First, HGECs were employed in our experiment; our results need to be verified in other basic endothelial cells. Second, how SETD8 and bach1 regulate Snail in high glucose-treated HGECs should be further investigated. Third, safe and effective SETD8 agonists that can be used in humans need to be further explored.

## Conclusion

In summary, our data indicated that during the development of DN, the expression of SETD8 was reduced, while the levels of ELK1 and bach1 were upregulated, thereby inducing EndMT in DN. Our data indicated that upregulation of bach1 expression played a key role in high glucose-induced EndMT in HGECs. Furthermore, SETD8 not only regulated the transcription of Snail directly but also cooperated with ELK1 to modulate bach1 expression, thus inducing hyperglycaemia-induced EndMT in DN.

## Supplementary Information

**Additional file 1: Table S1.** Primers used for the real-time RT-PCR analysis.

**Additional file 2: Figure S1.** Development of EndMT and increased expression of bach1 in DN. **a** Representative images of HE staining of DN rats and the control group (n = 10/group, scale bar: 20 μm). **b** Representative images of Masson’s trichrome staining of DN rats and the control group (n = 10/group, scale bar: 20 μm). **c**–**g** The IHC results of different genes in renal biopsy specimen of DN rats and the control group (n = 10/group, scale bar: 20 μm).

**Additional file 3: Figure S2.** Bach1 is required for EndMT in HGECs under hyperglycaemic conditions. **a** Western blot results of genes in different rat groups. **b**–**f** mRNA expression of genes in different rat groups (n = 5/group). **g** The result of Co-IP verified the connection between bach1 and SETD8. **h** Western blot results of genes in different cell groups. **i**–**m** mRNA expression of genes in different cell groups (n = 5/group). **n** SETD8 gathered at the Snail promoter region. **o** SETD8 and bach1 located at the same promoter region of Snail in HGECs. (Data are presented as the means ± standard deviation, *p < 0.05, **p < 0.01, ***p < 0.001, ****p < 0.0001, statistical analysis was carried out by a oneway ANOVA test).

**Additional file 4: Figure S3.** ELK1 participated in EndMT by augmenting bach1 expression in high glucose-cultured HGECs. **a** Immunostaining of ELK1 in the DN patients and the control group (n = 10/group, scale bar: 50 μm). **b** Western blot result of ELK1 in different rat groups. **c** mRNA expression of ELK1 in different rat groups (n = 5/group). **d** Immunostaining of ELK1 in the DN rats and the control group (n = 10/group, scale bar: 20 μm). (Data are presented as the means ± standard deviation, *p < 0.05, **p < 0.01, ***p < 0.001, ****p < 0.0001, statistical analysis was carried out by a oneway ANOVA test).

**Additional file 5: Figure S4.** ELK1 is related to SETD8. **a** ELK1 indirectly interacted with SETD8 (https://inbio-discover.intomics.com/map.html#search). **b** The enriched gene ontology (GO) terms (p < 0.05). The vertical axis in the graph represents the number of significant proteins. The horizontal axes represent the enriched GO terms. (BP: biological processes; MF: molecular functions; CC: cellular components). **c** Immunostaining of SETD8 in the DN patients and the control group (n = 10/group, scale bar: 50 μm). **d** Western blot result of SETD8 in different rat groups. **e** mRNA expression of STED8 in different rat groups (n = 5/group). **f** Immunostaining of SETD8 in the DN rats and the control group (n = 10/group, scale bar: 20 μm). (Data are presented as the means ± standard deviation, *p < 0.05, **p < 0.01, ***p < 0.001, ****p < 0.0001, statistical analysis was carried out by a oneway ANOVA test).

**Additional file 6: Figure S5.** Suppression of SETD8 regulated hyperglycaemia-induced EndMT by enhancing bach1 expression in HGECs. **a** Western blot results of genes in different cell groups. **b**–**g** mRNA expression of genes in different cell groups. (n = 5/group, data are presented as the means ± standard deviation, *p < 0.05, **p < 0.01, ***p < 0.001, ****p < 0.0001, statistical analysis was carried out by a oneway ANOVA test).

**Additional file 7: Figure S6.** Overexpression of SETD8 ameliorated the pathological process in rats with DN. **a** Overexpression of AVV-con, AVV-SETD8 in rat kidney were confirmed by immunofluorescence assay. **b**–**e** Immunostaining of different genes in kidney of rats with corresponding treatments (n = 10/group, data are presented as the means ± standard deviation,*p < 0.05, **p < 0.01, ***p < 0.001, ****p < 0.0001, statistical analysis was carried out by a one-way ANOVA test).

**Additional file 8: Figure S7.** Renal dysfunction in DN rats was improved by SETD8 overexpression. **a** Weight of rats in different groups. **b** Kidney weight of rats in different groups. **c** Fasting blood sugar (FBS) of rats in different groups. **d** Urine microprotein (UMP) of rats in different groups. **e** Creatinine (CREA) of rats in different groups. **f** Urine creatinine (UREA) of rats in different groups. (n = 10/group, data are presented as the means ± standard deviation, *p < 0.05, **p < 0.01, ***p < 0.001, ****p < 0.0001, statistical analysis was carried out by a one-way ANOVA test).

## Data Availability

The datasets used and/or analysed during the current study are available from the corresponding author on reasonable request.

## Abbreviations

DN: Diabetic nephropathy
EndMT: Endothelial-to-mesenchymal transition
HGECs: Human glomerular endothelial cells
H4K20me1: Histone H4 lysine20 methylation
bach1: Bric-a-brac/Tramtrack/Broad (BTB) and cap'n'collar (CNC) homology 1
ELK1: E26 transformation specific (ETS)-domain containing protein
SETD8: SET domain-containing protein 8
