# Supplementary material for: The SETD8/ELK1/bach1 complex regulates hyperglycaemia-mediated EndMT in diabetic nephropathy
Source: J Transl Med. 2022 Mar 29;20:147. doi: 10.1186/s12967-022-03352-4 (PMC8961497; doi:10.1186/s12967-022-03352-4)
Supplement: Supplementary file 1 — Additional file 1: Table S1. Primers used for the real-time RT-PCR analysis. [file 12967_2022_3352_MOESM1_ESM.docx]

| Species | RNA sequence |
| --- | --- |
| Human  β-actin  SETD8  ELK1  bach1  Snail  α-SMA  VIMENTIN  CD31      Rat  β-actin  SETD8  ELK1  bach1    Snail  α-SMA  VIMENTIN    CD31 | F 5’- CGGCTACAGCTTCACCACCAC -3’  R 5’- GCCATCTCTTGCTCGAAGTCCAG -3’  F 5’- TCCAGCAATCCTCCTCCTTCCTC -3’  R 5’- CCAGCCTAAGCAACAGATCCAGA -3’  F 5’- CCAAACCTGAAATCGGAAGA-3’  R 5’- CTCCTGTGGAGGGACTTCTG-3’  F 5’- AAGGAAATGCAAAAGCCTCA-3’  R 5’- TCCCCAGTACGGACTCTGTC-3’  F 5’- ACCCAGCACCATGAAGATCA-3’  R 5’- TTTGCGGTGGACAATGGAAG -3’  F 5’- ACCCAGCACCATGAAGATCA-3’  R 5’- TTTGCGGTGGACAATGGAAG -3’  F 5’- TACACAATTGCCTCTCCCCC -3’  R 5’-ACTCCTGTCTGAGATTACCCT -3’  F 5’- ACAGGACCGCGTTTTATCCTT -3’  R 5’- CCTTCCCAGTTCTGGGTTCTT -3’  F 5’- CTTCCAGCCTTCCTTCCTGG -3’  R 5’- GAGCCACCAATCCACACAGA -3’  F 5’- GCAGGAAGAGAACTCCGTCG -3’  R 5’- AGAATCACATGACGGGGGTG -3’  F 5’-AGCGGCCAGAAGTTTGTCTA-3’  R 5’- CTGTCATTCCTGCACCCTTT-3’  F 5’- GCATTTGGAACCGACAAGAT-3’  R 5’- GGGGTCTTTGCACTTTGTGT-3’  F 5’-CCTTTCTCCTGCTCCCACTG -3’  R 5’-CTCTTGGTGTTTGTGGAGCA -3’  F 5’- CATCATGCGTCTGGACTTGG -3’  R 5’- CCAGGGAAGAAGAGGAAGCA -3’  F 5’- GCCCCACCCTGAACCTAAAC -3’  R 5’- TGCCCTTCCCAACAATCACA -3’  F 5’- AGGCTGCCCTCAAACTCATC -3’  R 5’- CCCAACACGGATGCAAAAGG -3’ |

Table S1 Primers used for real-time RT-PCR analysis.
