# Supplementary figures and images for: The SETD8/ELK1/bach1 complex regulates hyperglycaemia-mediated EndMT in diabetic nephropathy
Source: J Transl Med. 2022 Mar 29;20:147. doi: 10.1186/s12967-022-03352-4 (PMC8961497; doi:10.1186/s12967-022-03352-4)

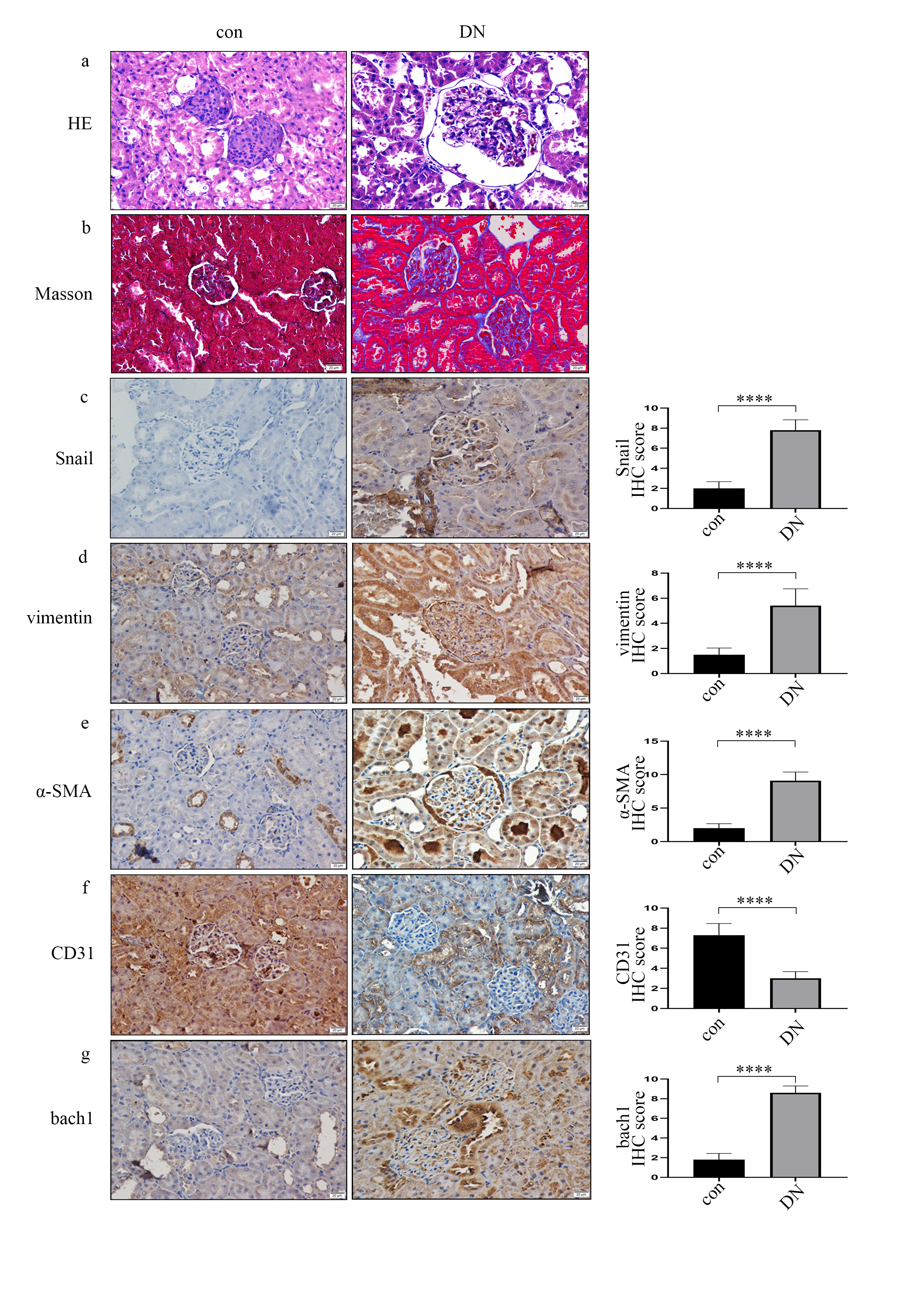

Supplement: Supplementary file 2 — Additional file 2: Figure S1. Development of EndMT and increased expression of bach1 in DN. a Representative images of HE staining of DN rats and the control group (n = 10/group, scale bar: 20 μm). b Representative images of Masson’s trichrome staining of DN rats and the control group (n = 10/group, scale bar: 20 μm). c–g The IHC results of different genes in renal biopsy specimen of DN rats and the control group (n = 10/group, scale bar: 20 μm). [file 12967_2022_3352_MOESM2_ESM.tif]

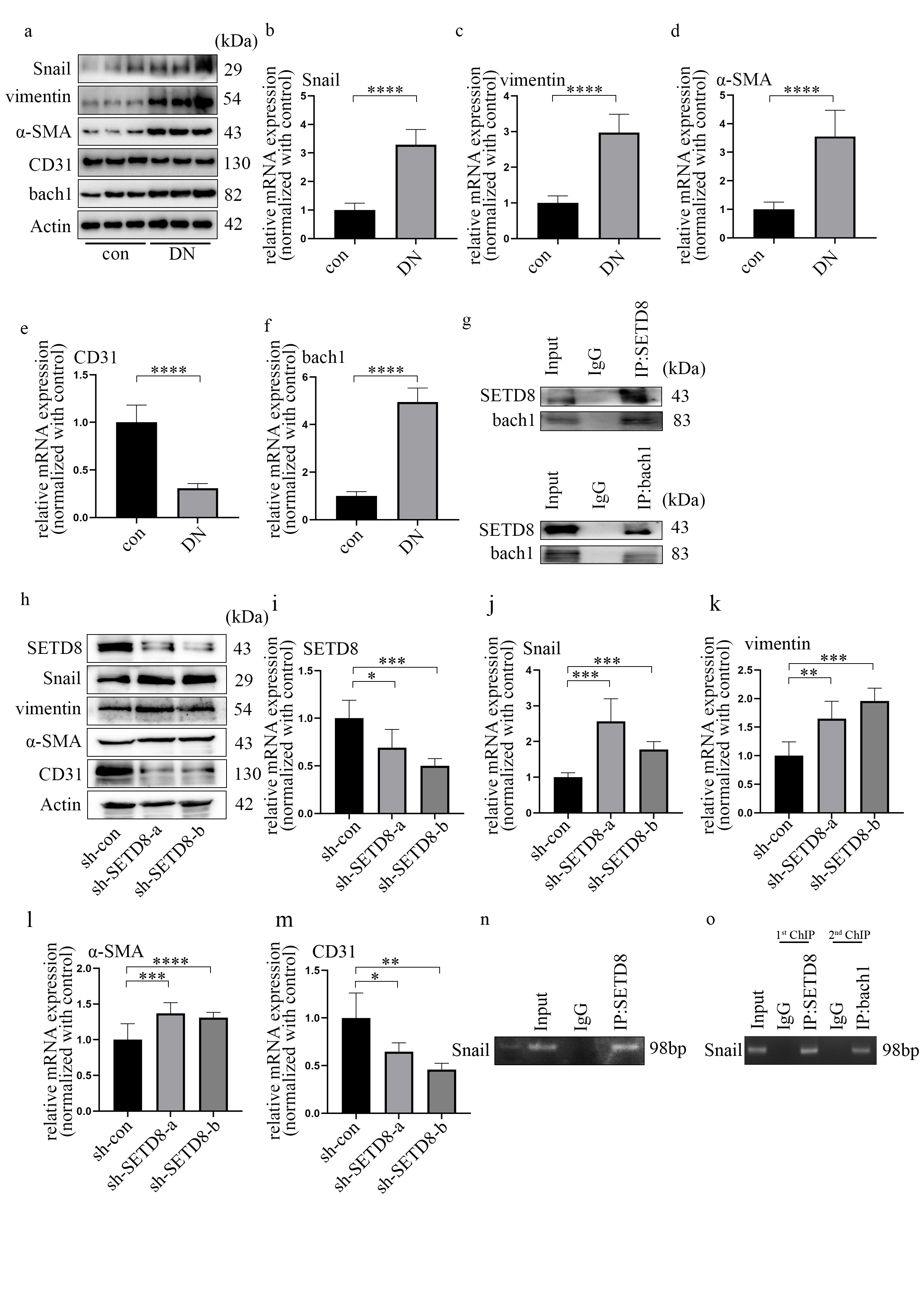

Supplement: Supplementary file 3 — Additional file 3: Figure S2. Bach1 is required for EndMT in HGECs under hyperglycaemic conditions. a Western blot results of genes in different rat groups. b–f mRNA expression of genes in different rat groups (n = 5/group). g The result of Co-IP verified the connection between bach1 and SETD8. h Western blot results of genes in different cell groups. i–m mRNA expression of genes in different cell groups (n = 5/group). n SETD8 gathered at the Snail promoter region. o SETD8 and bach1 located at the same promoter region of Snail in HGECs. (Data are presented as the means ± standard deviation, *p < 0.05, **p < 0.01, ***p < 0.001, ****p < 0.0001, statistical analysis was carried out by a oneway ANOVA test). [file 12967_2022_3352_MOESM3_ESM.tif]

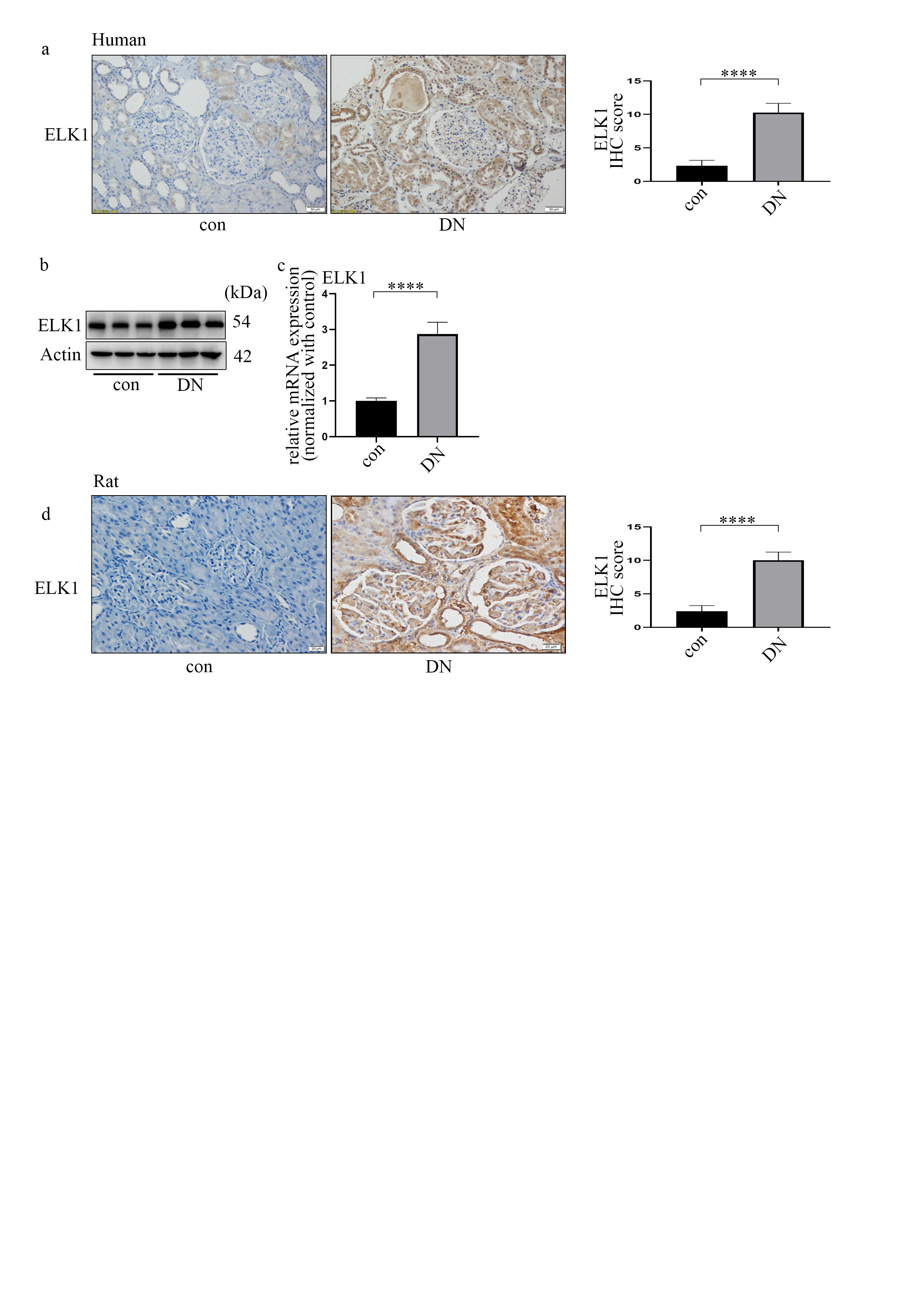

Supplement: Supplementary file 4 — Additional file 4: Figure S3. ELK1 participated in EndMT by augmenting bach1 expression in high glucose-cultured HGECs. a Immunostaining of ELK1 in the DN patients and the control group (n = 10/group, scale bar: 50 μm). b Western blot result of ELK1 in different rat groups. c mRNA expression of ELK1 in different rat groups (n = 5/group). d Immunostaining of ELK1 in the DN rats and the control group (n = 10/group, scale bar: 20 μm). (Data are presented as the means ± standard deviation, *p < 0.05, **p < 0.01, ***p < 0.001, ****p < 0.0001, statistical analysis was carried out by a oneway ANOVA test). [file 12967_2022_3352_MOESM4_ESM.tif]

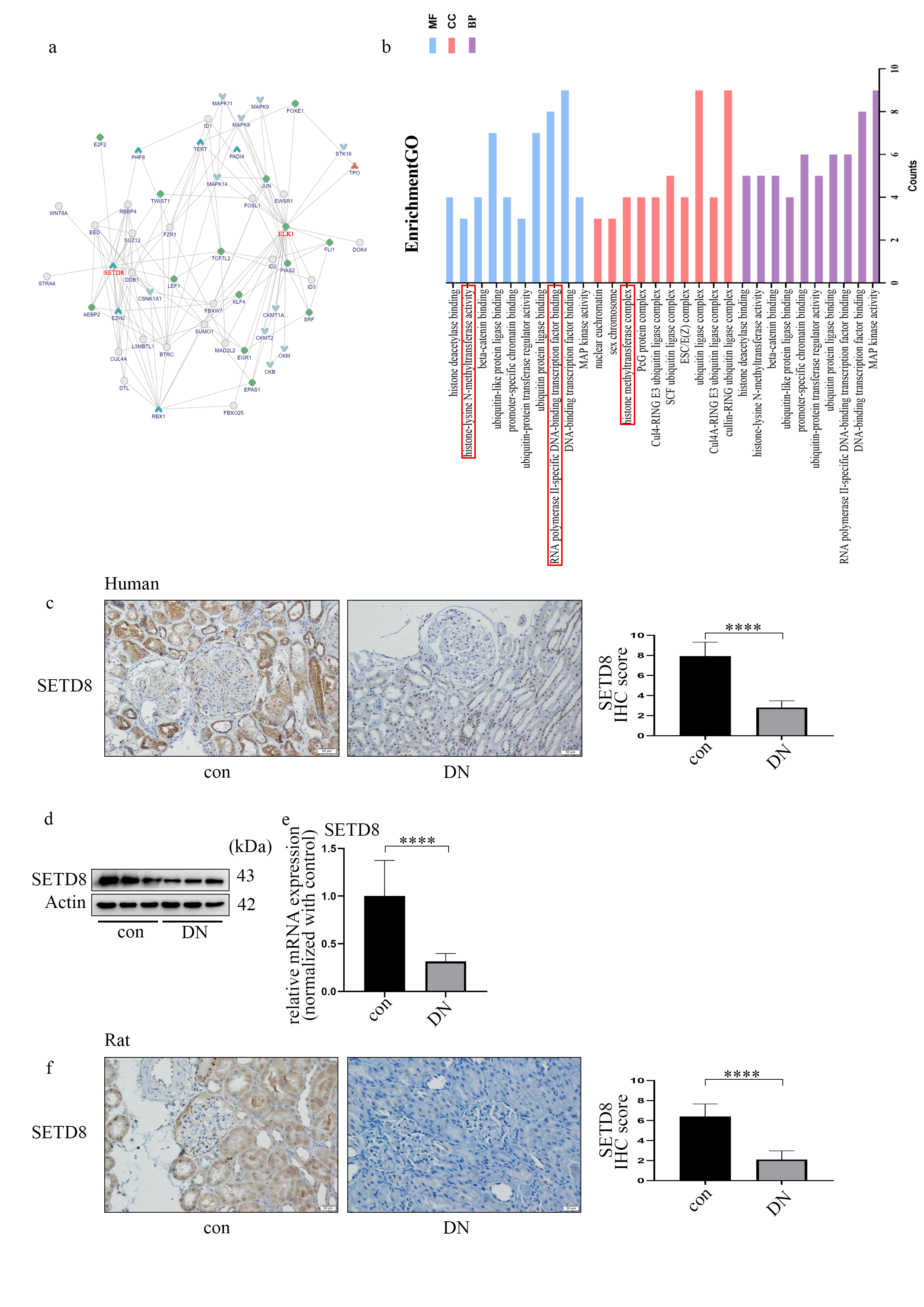

Supplement: Supplementary file 5 — Additional file 5: Figure S4. ELK1 is related to SETD8. a ELK1 indirectly interacted with SETD8 (https://inbio-discover.intomics.com/map.html#search). b The enriched gene ontology (GO) terms (p < 0.05). The vertical axis in the graph represents the number of significant proteins. The horizontal axes represent the enriched GO terms. (BP: biological processes; MF: molecular functions; CC: cellular components). c Immunostaining of SETD8 in the DN patients and the control group (n = 10/group, scale bar: 50 μm). d Western blot result of SETD8 in different rat groups. e mRNA expression of STED8 in different rat groups (n = 5/group). f Immunostaining of SETD8 in the DN rats and the control group (n = 10/group, scale bar: 20 μm). (Data are presented as the means ± standard deviation, *p < 0.05, **p < 0.01, ***p < 0.001, ****p < 0.0001, statistical analysis was carried out by a oneway ANOVA test). [file 12967_2022_3352_MOESM5_ESM.tif]

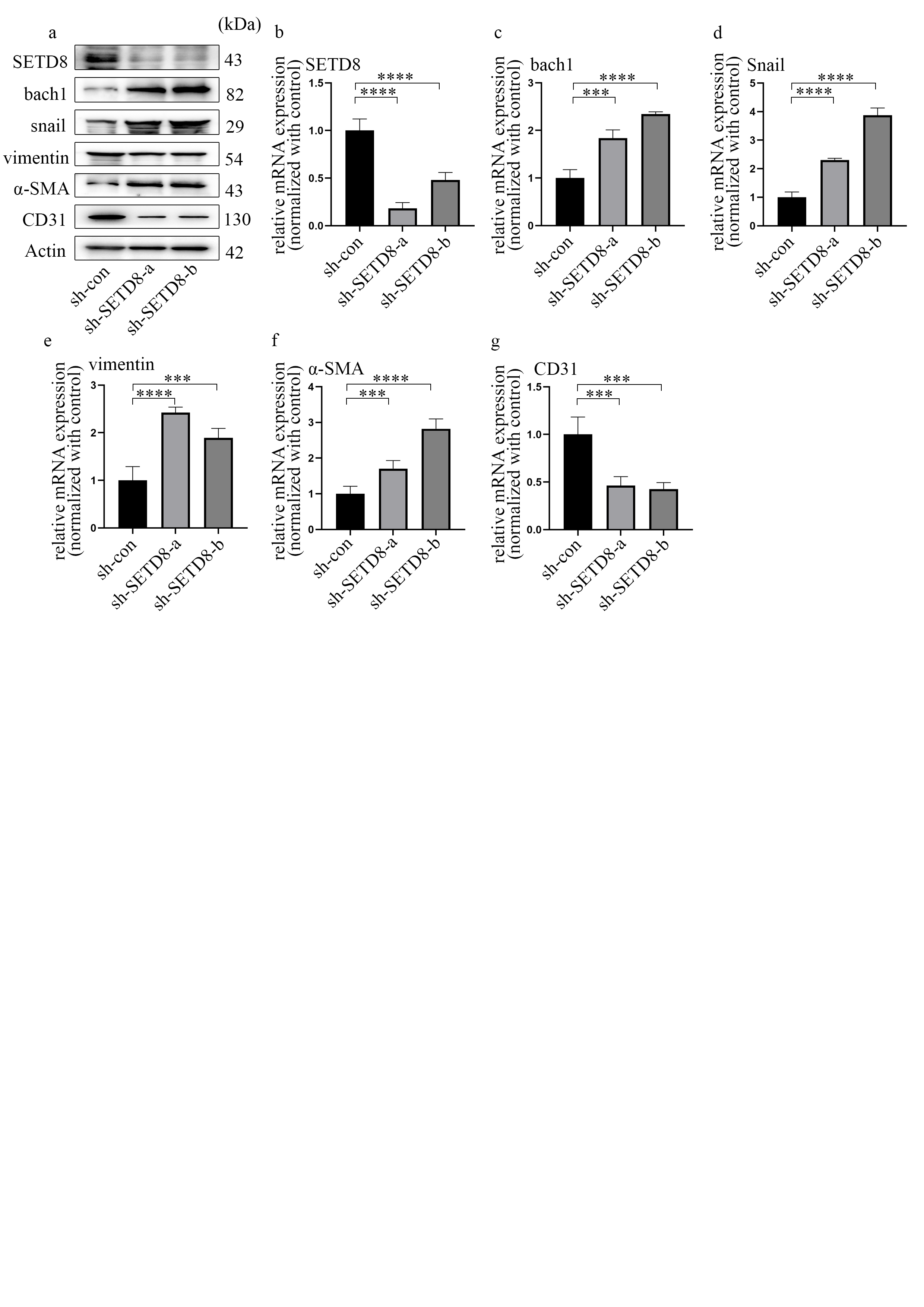

Supplement: Supplementary file 6 — Additional file 6: Figure S5. Suppression of SETD8 regulated hyperglycaemia-induced EndMT by enhancing bach1 expression in HGECs. a Western blot results of genes in different cell groups. b–g mRNA expression of genes in different cell groups. (n = 5/group, data are presented as the means ± standard deviation, *p < 0.05, **p < 0.01, ***p < 0.001, ****p < 0.0001, statistical analysis was carried out by a oneway ANOVA test). [file 12967_2022_3352_MOESM6_ESM.tif]

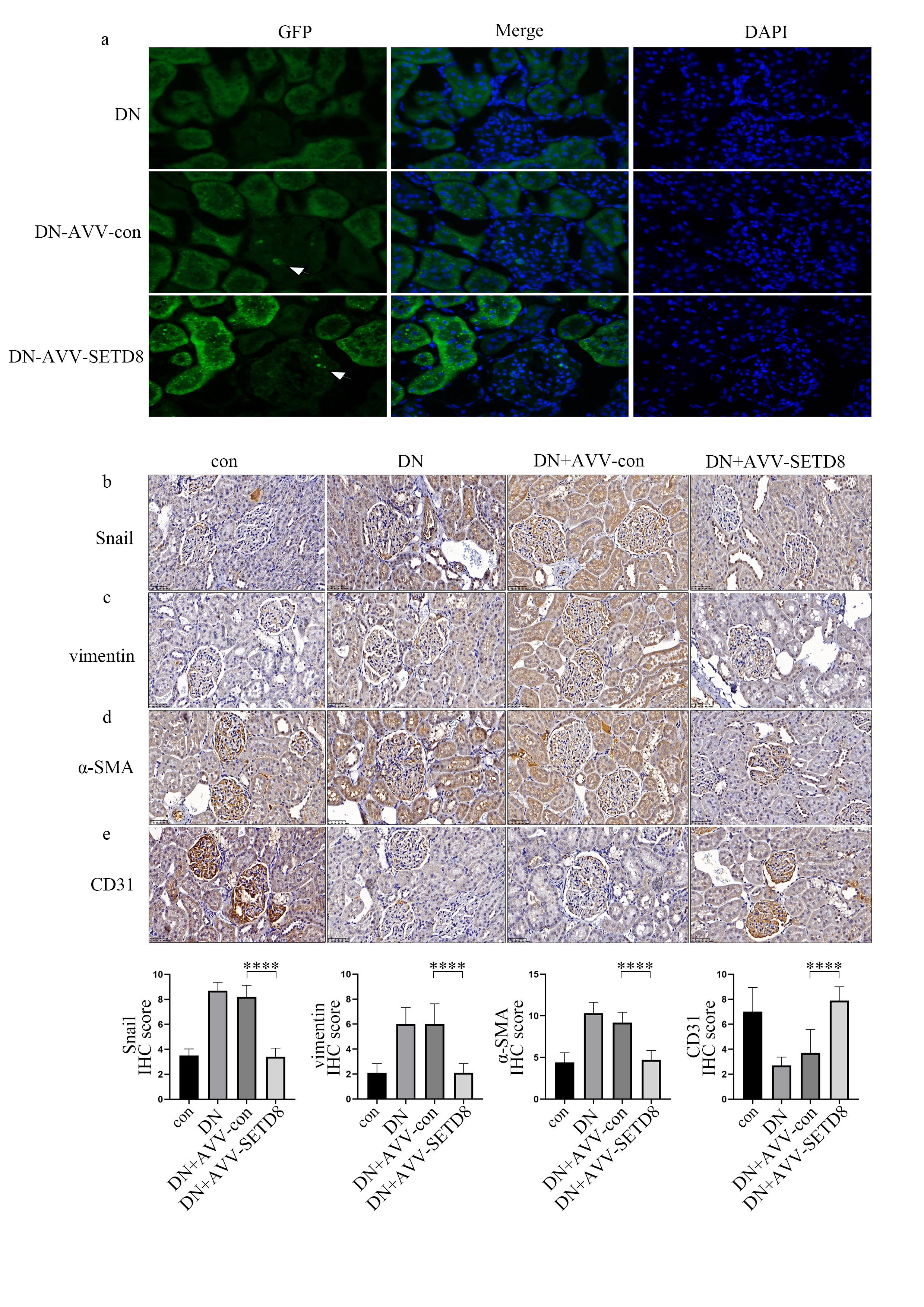

Supplement: Supplementary file 7 — Additional file 7: Figure S6. Overexpression of SETD8 ameliorated the pathological process in rats with DN. a Overexpression of AVV-con, AVV-SETD8 in rat kidney were confirmed by immunofluorescence assay. b–e Immunostaining of different genes in kidney of rats with corresponding treatments (n = 10/group, data are presented as the means ± standard deviation,*p < 0.05, **p < 0.01, ***p < 0.001, ****p < 0.0001, statistical analysis was carried out by a one-way ANOVA test). [file 12967_2022_3352_MOESM7_ESM.tif]

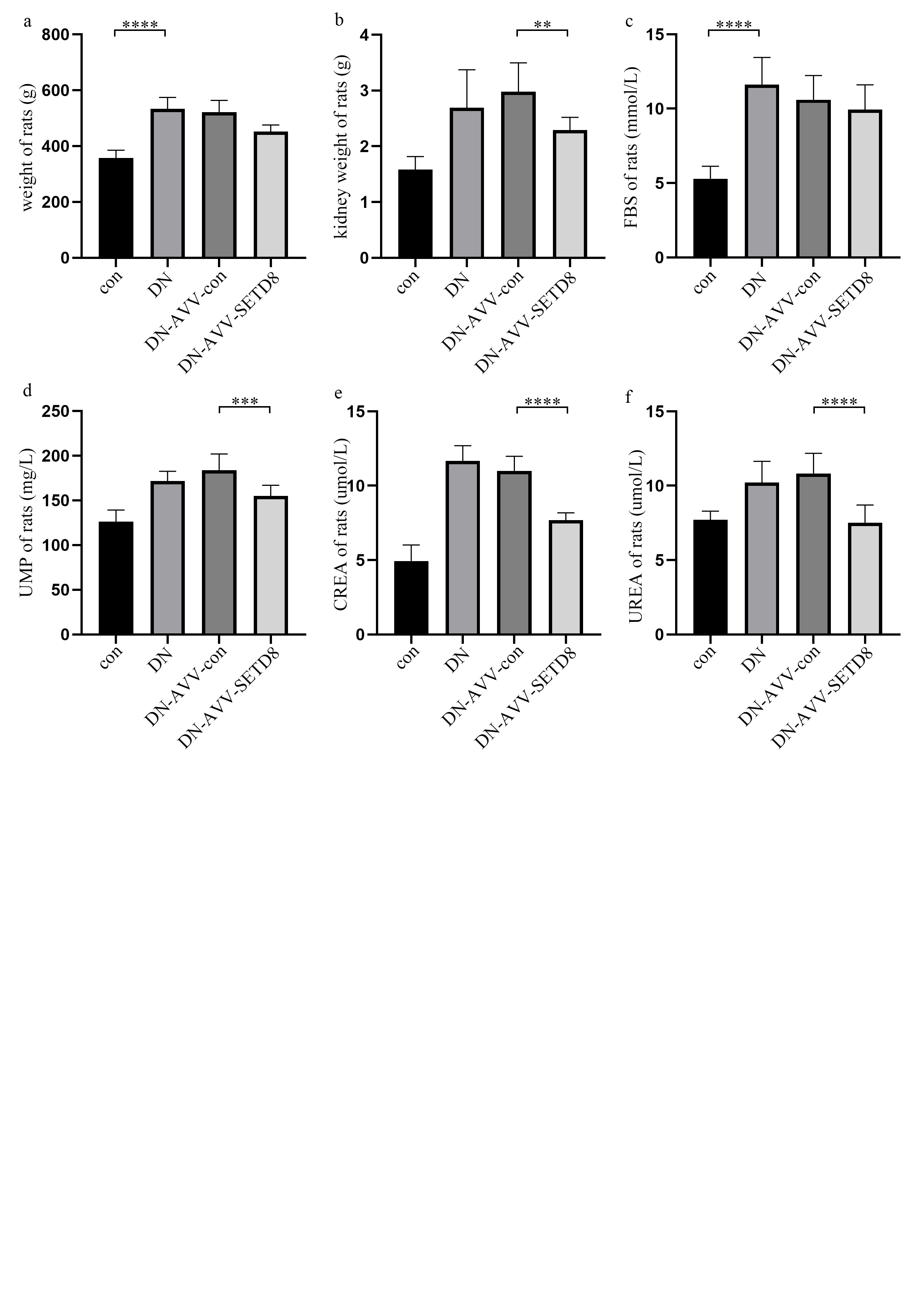

Supplement: Supplementary file 8 — Additional file 8: Figure S7. Renal dysfunction in DN rats was improved by SETD8 overexpression. a Weight of rats in different groups. b Kidney weight of rats in different groups. c Fasting blood sugar (FBS) of rats in different groups. d Urine microprotein (UMP) of rats in different groups. e Creatinine (CREA) of rats in different groups. f Urine creatinine (UREA) of rats in different groups. (n = 10/group, data are presented as the means ± standard deviation, *p < 0.05, **p < 0.01, ***p < 0.001, ****p < 0.0001, statistical analysis was carried out by a one-way ANOVA test). [file 12967_2022_3352_MOESM8_ESM.tif]
